# Fabrication of Chitosan/Graphene Oxide/PVA-Vanillin@TiO_2_ Composites for Anti-Inflammatory Drug Removal from Wastewater

**DOI:** 10.3390/nano16070414

**Published:** 2026-03-29

**Authors:** Anastasia D. Meretoudi, Athanasia K. Tolkou, Stavros G. Poulopoulos, Rigini M. Papi, Dimitra A. Lambropoulou, George Z. Kyzas

**Affiliations:** 1 Hephaestus Laboratory, School of Chemistry, Faculty of Sciences, Democritus University of Thrace, GR-65404 Kavala, Greece; ameretou@chem.duth.gr (A.D.M.); atolkou@chem.duth.gr (A.K.T.); 2 Chemical and Materials Engineering Department, School of Engineering and Digital Sciences, Environmental Science & Technology Group (ESTg), Nazarbayev University, Astana 010000, Kazakhstan; stavros.poulopoulos@nu.edu.kz; 3 Laboratory of Biochemistry, School of Chemistry, Aristotle University of Thessaloniki, GR-54124 Thessaloniki, Greece; rigini@chem.auth.gr; 4 Laboratory of Environmental Pollution Control, School of Chemistry, Aristotle University of Thessaloniki, GR-54124 Thessaloniki, Greece; dlambro@chem.auth.gr

**Keywords:** chitosan, vanillin, titanium dioxide, graphene oxide, NSAIDs, pharmaceuticals

## Abstract

In this work, three functionalized hybrid composites, CS/PVA-VAN, CS/PVA-VAN@TiO_2_ and CS/GO/PVA-VAN@TiO_2_, were synthesized and applied for adsorption evaluation on two common non-steroidal anti-inflammatory drugs, i.e., diclofenac (DCF) and ketoprofen (KTP). The structural and morphological characteristics of new composites were identified via Fourier-transform infrared spectroscopy (FTIR), scanning electron microscopy (SEM), X-ray diffraction (XRD) and BET techniques. BET analysis demonstrated that the CS/GO/PVA-Van@TiO_2_ composite has a surface area 64.86 m^2^/g, which is twice that of CS/PVA-Van. Moreover, adsorption evaluation was achieved at an optimum pH condition (pH 5.0) for both drugs. In addition, the kinetic data fitted better in a pseudo-second-order kinetic model, while the adsorption was heterogeneous and multilayer. The adsorption capacity of CS/GO/PVA-VAN@TiO_2_ was found to be 114.53 mg/g and 65.20 mg/g for diclofenac and ketoprofen, respectively. Thermodynamic analysis confirmed that the adsorption process was endothermic and spontaneous for all pollutants. Moreover, the kinetic swelling and stability studies demonstrated that graphene oxide contributed to improving the structural compactness and stability of composite. Finally, the adsorption performance of the optimal composite material was investigated in a binary system of non-steroidal anti-inflammatory drugs in various ratios.

## 1. Introduction

Non-steroidal anti-inflammatory drugs (NSAIDs) are a class of drugs used to treat various diseases such as inflammation in various arthritic and post-surgical conditions [1]. During the COVID-19 pandemic, the consumption of NSAIDs increased as various NSAIDs were used to treat different symptoms such as fever, muscle pain, etc. [2], affecting human health and ecosystem balance [3]. According to the literature, there are various common NSAIDs, such as diclofenac, ibuprofen, naproxen, ketoprofen etc., that are present in surface water and wastewater [4].

Diclofenac or 2-[2-(2,6-dichloroanilino) phenyl] acetic acid (DCF) is used widely due to its analgesic, anti-inflammatory, and antipyretic properties [5]. In spite of the therapeutic effect of DCF, many research groups have studied and documented toxic effects in mammals, such as neurotoxicity, cardiotoxicity, and bone marrow toxicity, due to the high level of DCF in surface water and wastewater [6]. Ketoprofen (KTP) and naproxen (NPX) belong in the propionic acid category [7] and they have similar chemical structures. These pharmaceutical compounds are recommended for the treatment of inflammatory and painful musculoskeletal disorders, such as osteoarthritis and rheumatoid arthritis [8]. However, a high level of KTP can cause toxicological effects in aquatic organisms [9].

In the literature, there are various different methods used to remove pharmaceutical compounds from wastewater; for example, adsorption, coagulation, flocculation and electrochemical methods [10]. However, a critical point for selecting the appropriate method is the combination of efficiency and cost-effective technique, such as adsorption [11].

One of the most common natural adsorbent materials is chitosan (CS). Chitosan is a natural polysaccharide (β1→4) with linked residues of N-acetyl-2 amino-2-deoxy-D-glucose (glucosamine) and 2-amino-2-deoxy-D-glucose (N-acetyl-glucosamine), used in biomedicine and environmental and agricultural applications as it is a biodegradable, non-toxic and low-cost material [12]. Due to the main functional groups –NH_2_ and –OH, chitosan is efficient in adsorption because it can increase intermolecular interaction with the pollutant. To improve the adsorption capacity and enhance the chemical stability, chitosan is modified with various modification techniques, such as grafting, crosslinking, etc. [13]. In the recent literature, vanillin, which is a green crosslinker, has been widely used due to its characteristics, i.e., its biocompatibility, non-toxicity, low cost, and antimicrobial and antioxidant properties [14].

Moreover, vanillin is a phenolic aldehyde and therefore a Schiff base is formed between the aldehyde group of vanillin and the amino group of chitosan. This modification increase the *π-π* interactions between the adsorbent material and the pharmaceutical compounds due to the aromatic system [15]. Therefore, vanillin offers an extra animo group that it is important for electrostatic interaction with the polymeric chain or with the pollutants. Furthermore, the addition of TiO_2_ nanomaterials, which are present in the polymeric matrix, enhances the adsorption efficiency of the pharmaceutical compounds. The TiO_2_ nanoparticles are incorporated primarily to enhance the porosity and structural integrity of the composite material, thereby increasing the available surface area for adsorption [16]. In this study, their photocatalytic activity is not exploited, as the current focus was to investigate their role as an adsorptive medium. To increase the chemical stability of the polymeric complex, PVA (polyvinyl alcohol) and graphene oxide (GO) were also used. According to Zataline et al. 2023, if chitosan is combined with PVA the adsorption properties and mechanical strength are increased. On the other hand, GO is an adsorbent material that widely used to remove emerging contaminants from wastewater [17] due to its properties such as its extended surface area and various functional groups (carboxyl, epoxy, hydroxyl groups) that interact with the pollutants.

Previous studies have demonstrated the potential of chitosan-based bio-nanocomposites for wastewater treatment. For instance, El-Aziz et al. 2023 synthesized superabsorbent bio-nanocomposites based on grafted chitosan loaded with GO/TiO_2_, reporting removal efficiencies of 79.5% and 78.8% for Pd^2+^ and BR46, respectively [18]. Similarly, Aricov and Ludmila 2025 indicated that vanillin-modified chitosan effectively removed the aromatic compound Bisphenol A (BPA) from an aqueous solution [19]. The new composite CS/GO/PVA-VAN@TiO_2_ exhibits a unique combination of polymeric and nanomaterial components, resulting in an expanded active surface area and an exceptionally high adsorption performance. Furthermore, its demonstrated reusability over multiple cycles highlights its functional stability and sustainability, making the material both economical and environmentally efficient. In contrast to previous systems, which exhibit limited adsorption capacity and lack evaluations of their reusability, CS/GO/PVA-VAN@TiO_2_ clearly overcomes these limitations. This characteristic positions the material as a pioneering, effective and sustainable solution for the removal of emerging contaminants from aquatic systems.

## 2. Materials and Methods

### 2.1. Materials

Chitosan (CS) of high molecular weight with a degree of deacetylation (DDA) higher than 75% and vanillin (VAN) were supplied from Sigma-Aldrich-Merck KGaA, Darmstadt, Germany. Polyvinyl alcohol (PVA) was obtained from Thermo Scientific (Waltham, MA, USA), and titanium dioxide was purchased from Nanografi Nano Technology (Ankara, Turkey). Acetic acid (≥99%) was obtained from Fisher Chemicals (Hampton, NH, USA); acetone (>99%) and glutaraldehyde (GLA) 50% in H_2_O were obtained from Sigma-Aldrich-Merck KGaA, Darmstadt, Germany. Diclofenac sodium was supplied by Tokyo Chemical Industry (Tokyo, Japan), while ketoprofen 2-(3-Benzoylphenyl) propanoic acid was purchased from BLD pharm (Shanghai, China).

### 2.2. Synthesis

The CS/PVA-VAN composite was synthesized and subsequently evaluated for its efficacy in removing diclofenac (DCF) and ketoprofen (KTP) from aqueous media. Initially, 0.5 g of polyvinyl alcohol (PVA) was dissolved in 98 mL of double-distilled water under continuous magnetic stirring at 80 °C for 1 h. Upon complete dissolution, the solution was allowed to cool to 60 °C. Separately, 0.2 g of vanillin (VAN) was dissolved in 10 mL of acetone, and this solution was added dropwise to the PVA solution under vigorous stirring to ensure homogeneity. Subsequently, 1 g of chitosan (CS) was incorporated into the mixture (Table 1). To facilitate crosslinking, 2 mL of acetic acid was introduced, followed by the addition of 1 mL of glutaraldehyde (GLA). VAN acts as the primary crosslinking agent in this system, reacting with the free amino groups of the polymer to form Schiff bases, thereby establishing the main crosslinked network that underpins the material’s structural cohesion. On the other hand, GLA was incorporated in only small amounts and serves a complementary role, primarily enhancing the initial stabilization of the network and reinforcing mechanical integrity. The reaction mixture was maintained under stirring at 60 °C for 4 h. The samples were then placed in the freezer for 24 h. The resulting composite was subjected to lyophilization for 48 h and further purified using a Soxhlet extractor with a 1:1 (*v*/*v*) acetone/water solvent system to eliminate the residual crosslinker, i.e., glutaraldehyde. The samples were dried at 60 °C for 24 h, resulting in the final composite for adsorption evaluation.

Regarding the synthesis of the CS/PVA-VAN@TiO_2_ composite, 0.2 g of titanium dioxide (TiO_2_) was added to the PVA solution prepared for the previous synthesis (of CS/PVA-VAN) and stirred for 30 min to ensure uniform dispersion. Following this step, chitosan (CS) was added to the mixture, and the synthesis proceeded as described.

**Figure 1:**
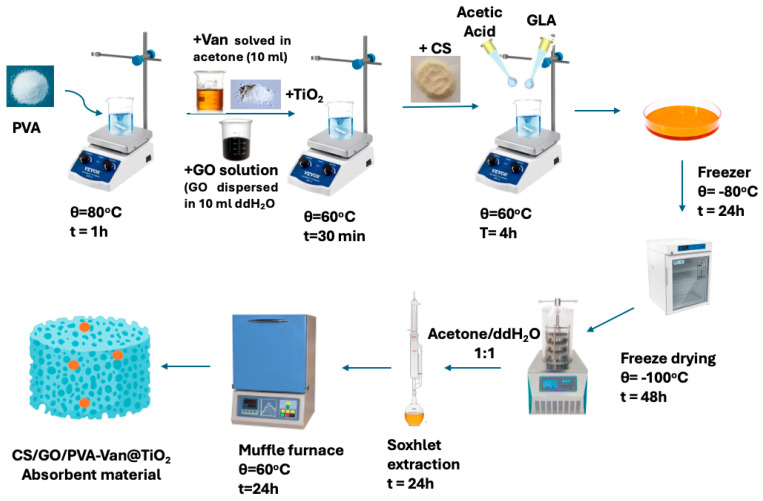
Experimental scheme of CS/GO/PVA-Van@TiO_2_ composite synthesis.

Concerning the synthesis of the CS/GO/PVA-VAN@TiO_2_ composite (Figure 1), GO was prepared in the laboratory according to the procedure reported by [20]. For this purpose, 0.01 g of GO was dispersed in 10 mL of double-distilled water and sonicated for 1 h at 30 °C. The resulting GO dispersion was then added to the PVA solution prepared for the previous synthesis (of CS/PVA-VAN@TiO_2_) and stirred for 1 h to ensure proper integration prior to the addition of CS.

### 2.3. Characterization Techniques

To provide a structural and physicochemical characterization of the prepared composites, various advanced techniques were used. Fourier-transform infrared (FTIR) spectroscopy was performed with a (ATR-FTIR, ZnSe) spectrometer (Perkin Elmer, New York, NY, USA) to identify the functional groups and chemical bonds. Crystallographic analysis was carried out using X-ray diffraction (XRD) with a BRUKER D8 FOCUS diffractometer (Karlsruhe, Germany). The surface morphology was examined via a JEOL JSM6390LV scanning electron microscope (SEM) (Tokyo, Japan). The specific surface area, pore size, and volume of the aerogels were determined by N_2_ adsorption with a NOVA 4200a (Quantachrome, Boynton Beach, FL, USA).

### 2.4. Adsorption Experiments

To determine the optimum conditions for adsorption, different experiments were carried out. Firstly, the effect of pH was studied in the range of 3.0–9.0 over a 24 h period at 303 K. The scope of this step was to determine the optimal pH value that would be kept constant for the next series of experiments. According to the literature [21,22], the initial concentration of pharmaceutical compounds, DCF and KTP, was 50 mg/L. For all adsorption experiments, the adsorbent mass applied was 1 g/L, which was chosen from the effect of adsorbent mass, which determined the lowest adsorption mass that can achieve the highest removal efficiency of NSAIDs. Furthermore, to determine the equilibrium time, the concentrations of the NSAIDs were measured at 5, 10, 15, 20, 30, 60, 90, 120, 180, 360 and 1440 min at 303 Κ. The contact-time study allowed us to understand the kind of sorption (physisorption or chemisorption) occurring between the adsorbent material and the pollutant. Moreover, isotherm studies were carried out at various initial concentrations of DCF (10–90 mg/L) and KTP (10–50 mg/L). Finally, thermodynamic analysis was performed at 303 K, 313 K and 323 K to determine if the adsorption was an endothermic or an endothermic phenomenon.

### 2.5. Statistical Analysis

Experiments were performed independently in triplicate for each condition. The presented values represent the mean of the three replicates. Additionally, variability among the measurements is indicated by error bars, as calculated by Equation (1) [23]:

(1) $$SE=\frac{SD\;}{\surd N}$$

where *SE* is standard error, *SD* is standard deviation and *N* is the number of replicates.

The results are presented graphically to convey both the central tendency of the data and the precision of the mean estimate for each condition.

### 2.6. Adsorption Evaluation

Evaluation of the effectiveness of the adsorbent materials was achieved with the determination of removal efficiency and adsorption capacity under various experimental conditions. The removal efficiency percentage was determined according to Equation (2):

(2) $$R\left(\%\right)=\frac{\left(C_{0}-C_{e}\right)}{C_{0}}100\%$$

where *R* is the removal percentage (%) and *C*_0_ and *C_e_* are the initial and equilibrium concentration of DCF and KTP (mg/L).

Moreover, Equation (3) describes the adsorption capacity:

(3) $$Q_{t}=\frac{\left(C_{0}-C_{e}\right)V}{m}$$

where *Q_t_* is the adsorption capacity (mg/g), *C*_0_ and *C_e_* are the initial and equilibrium concentration (mg/L), *m* (g) is the amount of adsorbent material, and *V* (L) is the volume of solution used in the experiment.

#### 2.6.1. Isotherm Studies

In the literature there are various equilibrium models which describe the interaction between absorbent materials and pollutants. The most common isotherm models are those of Langmuir [24] and Freundlich [25]. The Langmuir model illustrated a homogenous adsorption process where all of the adsorption sites are equivalent and each active site can interact with a pollutant molecule [26]. On the other hand, a heterogenous adsorption is described in the Freundlich model, which explains that adsorption is multilayer and all active sites require different energies for adsorption [27]. The Langmuir and Freundlich isotherm models are expressed in Equation (4) and Equation (5), respectively.

(4) $$Q_{e}=\frac{Q_{m}K_{L}C_{e}}{1+K_{L}C_{e}}$$

where *C_e_* is the equilibrium concentration of the pollutants (mg/L), *Q_m_* is the maximum sorption capacity (mg/g), and *K_L_* = Langmuir constant, in L/mg.

(5) $$Q_{e}=K_{f}C_{e}^{1/n}$$

where *n* = heterogeneity factor, and *K_F_* = Freundlich constant related to the adsorption capacity, in (mg/g)∙(L/mg)^1/n^.

In addition, as will emerge from the following experimental results, it is also necessary to apply the Langmuir–Freundlich (L-F) equation model (known as the Sips isotherm model [28]) to the data of this study as well. The Sips isotherm, a combination between the Langmuir and Freundlich models, is a model that studies three parameters to adjust the experimental results, and the relevant equation used is the following Equation (6):

(6) $$Q_{e}=\frac{{Q_{m}K}_{LF}C_{e}^{1/n}}{{1+K}_{LF}C_{e}^{1/n}}$$

where K_LF_ = equilibrium constant of heterogeneous surfaces (L/g), Q_e_ = adsorbed amount at equilibrium (mg/g), Q_m_ = maximum adsorption capacity (mg/g), C_e_ = adsorbate equilibrium concentration (mg/L) and n = heterogeneity parameter (0 < n < 1). Equation (5) is used for both high and low solution concentrations. In particular, at low concentrations, this isotherm follows the Freundlich isotherm and at high concentrations it follows the Langmuir isotherm, predicting monolayer adsorption capacity [29].

#### 2.6.2. Kinetic Studies

Pseudo-first-order (PFO) [30], pseudo-second-order (PSO) [31], and intraparticle diffusion (IPD) [32] models were chosen to fit the kinetic data, as they are the most commonly applied models. The PFO model refers to physisorption due to weak intermolecular interaction, such as Van der Waals interaction, electrostatic interaction, etc., which increases between the adsorbent and the pollutant [33]. The PSO model describes chemisorption, in particular the formation of new chemical bonds between the adsorbent and NSAIDs [34]. Equations (7) and (8) express the PFO and PSO model, respectively, and intraparticle diffusion (IPD) is also described (Equation (9)). The adsorption process is best explained by the model with the highest correlation coefficient (*R*^2^):

(7) $$Q_{t}=Q_{e}(1-e^{-K_{1}t})$$

(8) $$Q_{t}=\frac{{Q_{e}}^{2}K_{2}t}{\left(1+Q_{e}K_{2}t\right)}$$

where *Q_e_* and *Q_t_* (mg/g) are the adsorption capacities at equilibrium and at time *t*, respectively, and *K*_1_ (1/min) and *K*_2_ (g/mg·min) are the first- and second-order rate constants.

(9) $$Q_{t}=K_{IPD}t^{0.5}+C$$

K_IPD_ is the rate constant (mg/g min0.5), while the constant C (mg/g) reflects the thickness of the boundary layer.

#### 2.6.3. Thermodynamic Study

To understand the nature of the adsorption mechanism, it is necessary to determine the thermodynamic parameters (to determine if adsorption is spontaneous or not). The change in Gibbs’s free energy is shown by Equations (10) and (11) [35]:

(10) $${\Delta G}^{0}=-RTln(K_{c})$$

(11) $${\Delta G}^{0}={\Delta H}^{0}-{T\Delta S}^{0}$$

where *R* is the gas constant (8.314 J/mol·K), *T* represents the temperature (K), and *K_c_* is the thermodynamic constant.

Moreover, the thermodynamic parameters related to adsorption include enthalpy change (Δ*H*^0^) and entropy change (Δ*S*^0^) and are described by Equations (12) and (13) [36].

(12) $${Κ}_{C}=\frac{Q_{e}}{C_{e}}$$

(13) $$ln\left({Κ}_{c}\right)=\left(-\frac{{\Delta H}^{0}}{RT}\right)+\frac{{\Delta S}^{0}}{R}$$

### 2.7. Effect of Mass

The adsorption experiment for the effect of the adsorbent’s mass was carried out under the following conditions to determine the influence of the initial adsorbent quantity on equilibrium. Specifically, the range of adsorbent mass was 0.2–1.5 g/L at optimum pH value, for 180 min at 303 K. The process was repeated 2 more times, such as in all batch experiments.

### 2.8. Regeneration

In recent decades, the critical point between the academic world and industry is the reuse of absorbent material for environmental and economic benefit [37]. The reusability of all new composites was tested in batch experiments using a desorbing agent. According to the literature, during desorption, each of the adsorbent materials was processed with a 0.01 M NaOH solution (pH 12) and then washed repeatedly until a neutral pH was obtained [38]. In this way, the pharmaceutical compound can be removed from the polymeric matrix of the adsorbent material and can then be reused in the next regeneration cycle.

### 2.9. Swelling

The swelling ratio was measured using distilled water at an optimum pH value of 5.0 for the DCF and KTP solutions. Briefly, CS/PVA-VAN, CS/PVA-VAN@TiO_2_ and CS/GO/PVA-VAN@TiO_2_ were weighed (*W*_1_) (g) and placed in solution. An 0.05 g amount of adsorbent was added to 50 mL of each solution. Then, measurements were taken at specific time intervals (5, 10, 15, 30, 45, 60, 90 and 120 min) until a stable weight was achieved. Using filter paper, the materials were blotted to remove excess surface water and then weighed (*W*_2_) (g) until a constant weight was achieved. The swelling percentage for each composite was calculated at equilibrium using the following Equation (14) [39].

(14) $$Swelling\;(\%)=\frac{W_{2}-W_{1}}{W_{1}}$$

where *W*_1_ is the initial and *W*_2_ the final amount of adsorbent dosage, respectively.

### 2.10. Stability Study

Adsorbent stability was evaluated in double-distilled water at various pH values (3.0, 5.0, 7.0, 9.0 and 11), which were adjusted using HCl and NaOH, respectively. In this experiment, 0.05 g (*W*_1_) of each adsorbent material was added to 50 mL of each solution for 24 h. Thereafter, the adsorbents were removed from solution and were placed in an oven at 60 °C until they completely dried (*W*_2_) (g). Finally, the composites were weighted and the mass loss was calculated using Equation (15) [40].

(15) $$Mass\;loss\;\left(\%\right)=\frac{W_{2}}{W_{1}}100\%$$

## 3. Results and Discussion

### 3.1. Characterization

#### 3.1.1. FTIR

**Figure 2:**
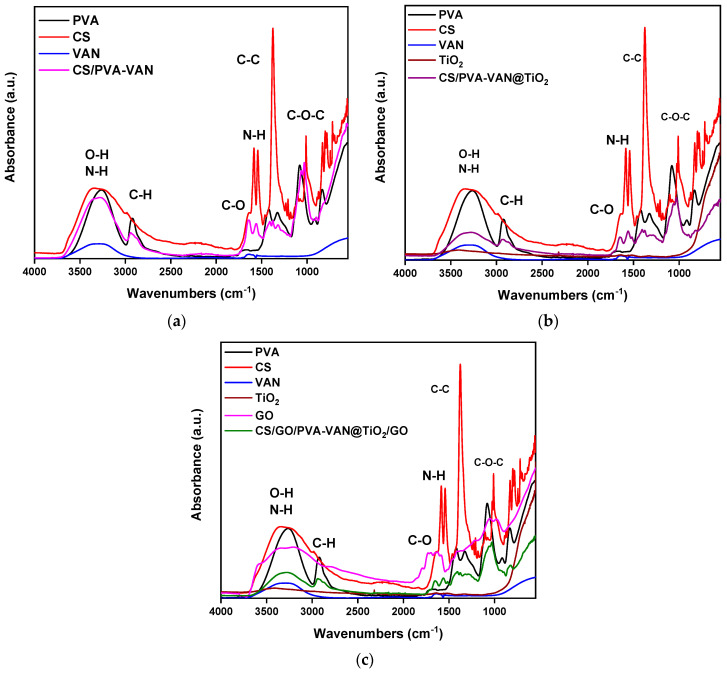
FTIR spectra of (**a**) CS/PVA-VAN, (**b**) CS/PVA-VAN@TiO_2_ and (**c**) CS/GO/PVA-VAN@TiO_2_ in comparison to net materials, before adsorption of DCF and KT.

Figure 2 presents the FTIR spectrum of CS/PVA-VAN, CS/PVA-VAN@TiO_2_, and CS/GO/PVA-VAN@TiO_2_. Firstly, the characteristic peaks of O–H and N–H are depicted at 3000–3500 cm^−1^ [41], while the vibration band of C–H is shown at 2941 cm^−1^ [42]. The stretching bands of C–C and C–O–C appeared at 1398 cm^−1^ [43] and 1020 cm^−1^ [44], respectively. In three new composite adsorbent materials, the vibrations of the N-H bond are shown at different peaks, depending on the material [45]. Specifically, the band N–H appeared at 1553 cm^−1^, 1562 cm^−1^ and 1563 cm^−1^ for CS/PVA-VAN, CS/PVA-VAN@TiO_2_ and CS/GO/PVA-VAN@TiO_2_, respectively. This peak displacement is due to intermolecular interactions which take place between the polymeric matrix and newly added chemical compounds, such as TiO_2_ and GO [46].

The FTIR spectra of the composite adsorbent materials shows significant shifts in almost all of the characteristic functional group peaks (Figure 2). More specifically, the C–H bond vibration appears at 2941, 2930, and 2935 cm^−1^ for the materials CS/PVA-VAN (Figure 2a), CS/PVA-VAN@TiO_2_ (Figure 2b), and CS/GO/PVA-VAN@TiO_2_ (Figure 2c), respectively, while for neat chitosan (CS), it appeared at the lower wavenumber of 2981 cm^−1^. This decrease in vibration is attributed to changes in the polarity of the environment [47]. In the CS spectra, the amide bond vibration appeared at 1587 cm^−1^, while in the CS/PVA-VAN composite it appeared at 1553 cm^−1^. This shift is attributed to the formation of a Schiff base between the amino group of CS and the aldehyde group of vanillin [48]. In the spectra of CSPVA-VAN@TiO_2_ and CS/GO/PVA-VAN@TiO_2_, broad absorption peaks showed in the regions of 1554–1564 and 1569–1579 cm^−1^, respectively, which are assigned to intermolecular interactions such as hydrogen bonding [49] and electrostatic interactions [50], due to the addition of TiO_2_ and GO into the polymeric matrix. Furthermore, the C=O bond in chitosan presented at 1658 cm^−1^, whereas in the composite materials, CS/PVA-Van, CS/PVA-VAN@TiO_2_ and CS/PVA-VAN@TiO_2_, it appeared at 1644, 1645, and 1643 cm^−1^, respectively. The decrease in vibration confirmed the formation of hydrogen bonds in the composites [51]. Overall, FTIR analysis verified the successful incorporation of the additives into the polymer matrix through Schiff base formation, hydrogen bonding, and electrostatic interactions.

**Figure 3:**
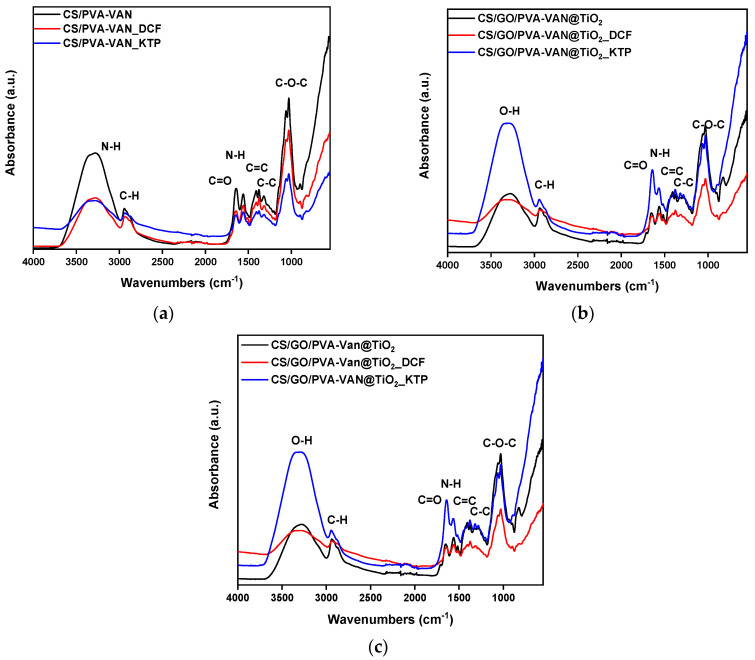
FTIR spectra of (**a**) CS/PVA-VAN, (**b**) CS/PVA-VAN@TiO_2_ and (**c**) CS/GO/PVA-VAN@TiO_2_ after adsorption of DCF and KTP.

Comparative analysis of the FTIR spectra obtained before and after the adsorption process was performed to identify spectral shifts or alterations indicating specific functional groups in the adsorption mechanism. Moreover, Figure 3 presents the FTIR spectra after the adsorption of the pharmaceutical compounds. As illustrated, in the CS/PVA-VAN spectra (Figure 3a), the vibration of C–C bonds appeared at 1383 cm^−1^ before adsorption, while after the adsorption of DCF and KTP it shifted to 1373 cm^−1^. This shift indicated the presence of π–π stacking interactions between the adsorbent and the pollutants [52]. Furthermore, in the CS/PVA-VAN@TiO_2_ spectra (Figure 3b), a shift is observed from 1650 to 1643 and 1646 cm^−1^ after the adsorption of DCF and KTP, respectively. This shift further supported the occurrence of interactions between the functional groups of the adsorbent and the pharmaceutical molecules.

After the adsorption of DCF and KTP onto the CS/GO/PVA-VAN@TiO_2_ composite (Figure 3c), distinct shifts were observed in the characteristic FTIR bands. The C=O stretching vibration, initially at 1648 cm^−1^, shifted to 1638 cm^−1^ for DCF and KTP. Simultaneously, the N-H stretching vibration decreased from 1403 cm^−1^ to 1372 cm^−1^. These observed shifts indicate the involvement of intermolecular interactions in the adsorption mechanism, such as hydrogen bonding, but the dominant contribution is π-π stacking interactions between the aromatic groups of the drugs and π-electron network of GO. Furthermore, hydrogen bonding also appears to play a role in stabilizing the adsorption process, particularly through interaction between the -OH and -NH_2_ groups of the matrix and polar functional groups of the drugs. Additionally, small shifts are observed in the C=C bond peaks, while no new peaks appear in the 1500–1600 cm^−1^ region. These shifts indirectly support the presence of π-π interactions between the substrate and the aromatic ring of the NSAIDs, without generating new distinct vibrations. This dominant adsorption mechanism, also reported for other CS–biochar composites, involves both non-covalent, Van der Waals interaction between the CS chain and biochar surface [53].

#### 3.1.2. XRD

**Figure 4:**
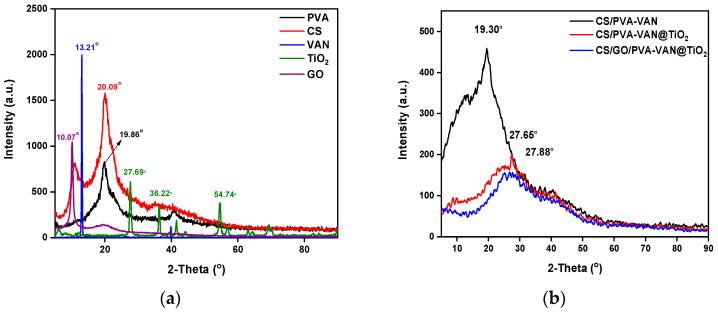
XRD pattern of (**a**) PVA, CS, VAN, TiO_2_ and GO and (**b**) CS/PVA-VAN, CS/PVA-VAN@TiO_2_ and CS/GO/PVA-VAN@TiO_2_.

XRD analysis (Figure 4a) of the individual components shows distinct differences in crystallinity that influence the structure and function of the composite. Initially, PVA and CS exhibit semi-crystalline characteristics at 19.85° and 20.09°, typical of flexible polymer matrices that promote homogeneous aerogel formation [54]. In addition, TiO_2_ exhibits multiple diffraction peaks at 27.66°, 36.22° and 54.47°. These peaks indicate the presence of more ordered domains that provide structural support [55]. However, VAN and GO show low-angle reflections at 13.21° and 10.07°. This suggests layered structures with large lattice spacings [56]. Such structures can enhance adsorption through hydrogen bonding, electrostatic interaction and increased surface area [57].

Additionally, the XRD patterns of the composite material reveal a noticeable decrease in the intensity and slight broadening of the characteristic peaks compared to the neat TiO_2_ and GO, indicating a reduction in crystallinity. The main diffraction peaks corresponding to TiO_2_ are still present, confirming the retention of its crystal structure, while the broadening suggests smaller crystallite sizes or increased structural disorder due to incorporation of GO. Moreover, no new peaks were observed, implying the absence of new crystalline phases. These changes suggest that the addition of TiO_2_ and GO affects the crystallinity and structural order of the composite.

#### 3.1.3. SEM Analysis

**Figure 5:**
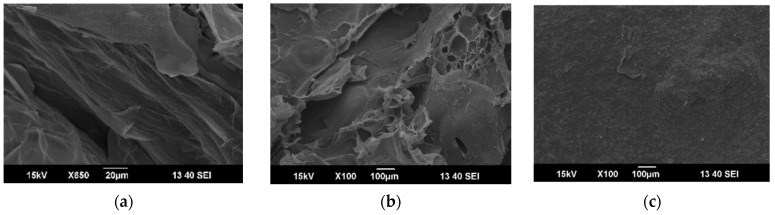
SEM images of (**a**) CS/PVA-VAN, (**b**) CS/PVA-VAN@TiO_2_ and (**c**) CS/GO/PVA-VAN@TiO_2_.

The morphology of CS/PVA-VAN, CS/PVA-VAN@TiO_2_ and CS/GO/PVA-VAN@TiO_2_ was assessed via SEM technique. The SEM image of the CS/PVA-VAN aerogel (Figure 5a) showed a relatively smooth and homogeneous surface without visible pores or cracks. This uniform morphology indicated complete miscibility and compatibility between PVA and chitosan, due to intermolecular interactions (as explained in Section 3.1.1). Moreover, Figure 5b presented the SEM image of CS/PVA-VAN@TiO_2_. The surface exhibited consistent roughness and integrity due to the complete incorporation of TiO_2_ into the polymeric matrix. Therefore, the SEM images of the CS/GO/PVA-VAN@TiO_2_ nanocomposite (Figure 5c) revealed a more compact and uniform structure. This phenomenon indicated strong interfacial interactions between GO and the polymer matrix, which enhanced internal cohesion [58]. A well-dispersed GO phase is crucial for improving the aerogels’ mechanical and structural properties [59].

#### 3.1.4. BET Analysis

**Figure 6:**
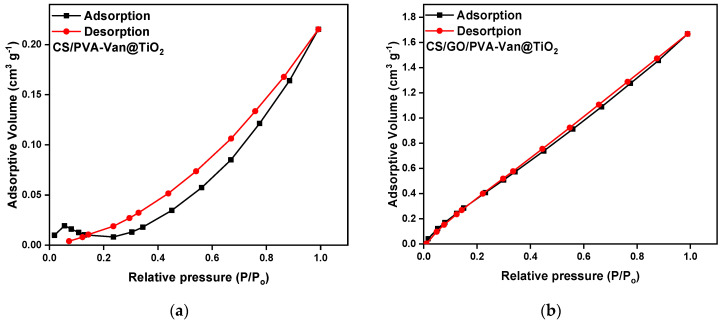
BET analysis of (**a**) CS/PVA-Van@TiO_2_ and (**b**) CS/GO/PVA-Van@TiO_2_.

The BET analysis of the two adsorbent materials reveals significant differences in pore structure and surface area (Figure 6). Initially, CS/PVA-Van@TiO_2_ exhibited a specific surface area of 32.46 m^2^/g, a pore volume of 0.029 cm^3^/g and a very high average pore diameter of 161.18 nm (Table 2), indicating a primarily microporous material with a limited available surface area for the adsorption of small molecules [60]. However, with the addition of GO in CS/GO/PVA-Van@TiO_2_, the specific surface area doubled to 64.86 m^2^/g and the pore volume increased to 0.060 cm^3^/g, while the average pore diameter decreased to 2.016 nm, suggesting a transformation to a micro–mesoporous structure [61]. The notable increase in specific surface area upon the addition of 0.01 g GO indicates that even trace amounts can effectively enhance the dispersion of TiO_2_ particles and influence the polymer matrix morphology. Moreover, the functional group of GO may interact with polymer chain, creating an extended porous structure while simultaneously reducing particle aggregation [62]. Consequently, this modification significantly improved the adsorption capacity and the availability of active sites [63], making the CS/GO/PVA-Van@TiO_2_ more suitable for the adsorption of small organic pollutants. Overall, the incorporation of GO improves both pore geometry and specific surface area, thereby improving the performance of the adsorption process.

### 3.2. Adsorption Evaluation

#### 3.2.1. Effect of pH—Adsorption Mechanism

The effect of pH is a critical factor in all batch experiments, as the pH value influences all other aspects of adsorption evaluation. In this experimental process, the removal efficiency (%) of DCF and KTP using the newly developed composite adsorbent materials was tested at pH values of 3.0, 5.0, 7.0, 9.0, and 11.0. The adsorbent mass was 1 g/L at 303 K. The aim was to determine the optimum pH that maximizes removal efficiency, considering that DCF and KTP have different pKa values (4.71 [64] and 4.76 [65], respectively), as well as different charge characteristics across the pH spectrum.

**Figure 7:**
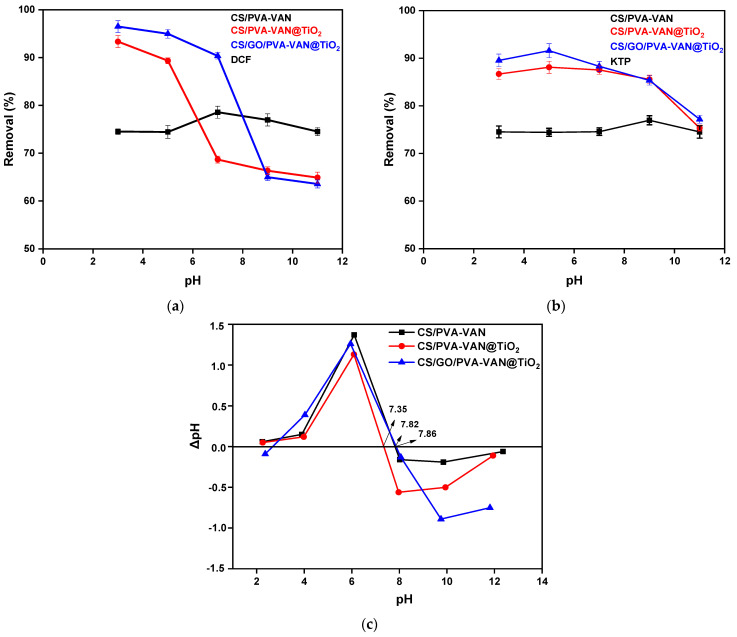
Effect of pH on the adsorption of (**a**) DCF and (**b**) KTP from CS/PVA-VAN, CS/PVA-VAN@TiO_2_ and CS/GO/PVA-VAN@TiO_2_ and (**c**) the pH_pzc_ of adsorbent materials. (C_0_ = 50 mg/L, dose = 1 g/L, T = 303 K, t = 24 h).

As presented in Figure 7c, the CS/GO/PVA-VAN@TiO_2_ composite exhibited the highest percentages in all examined NSAIDs compared to CS/PVA-VAN (Figure 7a) and CS/PVA-VAN@TiO_2_ (Figure 7b). Additionally, diagrams showed that the highest removal efficiency is achieved at pH 3.0 and 5.0. However, it is known that the NSAIDs hydrolyze at a lower pH value [66] and for this reason in this study the optimal pH value chosen is pH 5.0 (and not 3.0). In neutral and basic conditions, the removal efficiency decreased for all composites. Specifically, at pH 5.0, CS/GO/PVA-VAN@TiO_2_ removed 95% and 86% of DCF and KTP, respectively, while CS/PVA-VAN absorbed less than 80% of each pollutant. Therefore, CS/GO/PVA-VAN@TiO_2_ is identified as the most effective adsorbent for NSAID removal under the studied conditions.

**Figure 8:**
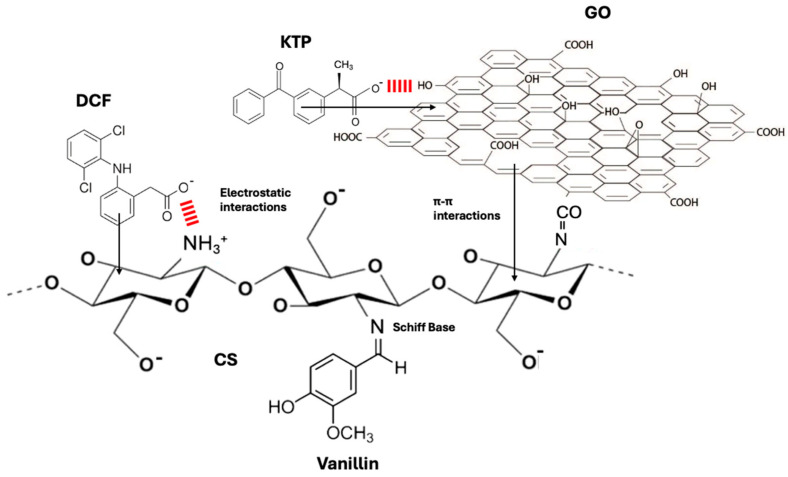
Schematic illustration of the proposed adsorption mechanism of DCF and KTP onto the optimum CS/GO/PVA-VAN@TiO_2_ composite.

Moreover, the point of zero charge (pH_pzc_) of the composite material was determined by the pH drift method [67] and is shown in Figure 7c. After calculation, the relative pH_pzc_ values for CS/PVA-VAN, CS/PVA-VAN@TiO_2_ and CS/GO/PVA-VAN@TiO_2_ were found to be 7.35, 7.82 and 7.86, respectively. Moreover, pH_pzc_ was important to describe the surface interaction between the adsorbent material and pollutant. Particularly, when pH < pH_pzc_, the adsorbent surface is positively charged, while when pH > pH_pzc_ it is negatively charged [68]. As previously mentioned, the optimum pH value for DCF and KTP removal was 5.0. This suggests that electrostatic interaction increased between the positive surface of the adsorbent and the negatively charged NSAIDs. Specifically, CS/GO/PVA-VAN@TiO_2_, due to the GO, offered various functional groups such as amino groups or carboxyl, hydroxyl and epoxy groups [69], which in low-pH conditions protonated and formed electrostatic interactions with the ionized structure of the NSAIDs (pH > pKa of DCF and KTP). According to the literature, π-π interactions between the aromatic rings of pollutants and carbon-based adsorbents enhance the adsorption efficiency, explaining the higher removal observed for CS/GO/PVA-Van_TiO_2_ [70]. These kinds of interactions with pharmaceutical compounds are responsible for the removal efficiency of CS/PVA-VAN. Figure 8 presents the proposed mechanism that may occur on the surface of the optimum CS/GO/PVA-VAN@TiO_2_ composite.

#### 3.2.2. Effect of Contact Time

**Figure 9:**
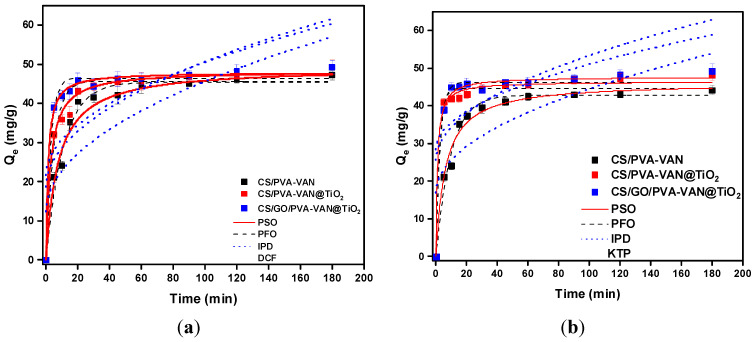
Effect of contact time on the adsorption of (**a**) DCF and (**b**) KTP for CS/PVA-VAN, CS/PVA-VAN@TiO_2_ and CS/GO/PVA-VAN@TiO_2_. (C_0_ = 50 mg/L, pH = 5.0, T = 303 K, t = 0–180 min).

Another important parameter for adsorption evaluation is the effect of contact time because it gives information about the equilibrium time and the adsorption type. In this study, each adsorbent material (30 mg, V = 30 mL equal to 1 g/L) was tested at pH 5.0 and 303 K for different contact times ranging from 5 to 180 min to observe how adsorption changes over time. Figure 9 presents the effect of contact time for all of the new composite materials in DCF and KTP solutions.

During the initial minutes of the adsorption process, a sharp increase in the adsorbent’s capacity is observed, which subsequently stabilizes, and equilibrium was reached at 45 min for DCF and at 60 min for KTP. The rapid initial adsorption in the active sites is followed by a slower stage governed by the intraparticle diffusion of the DCF and KTP molecules. This phenomenon suggests that the adsorption process is controlled by both chemisorption and diffusion-limited mechanisms.

Kinetic studies constitute a critical aspect in elucidating the adsorption process. The experimental data were analyzed using the PFO and PSO models, those most applied in adsorption research, while the IPD model was used to evaluate whether intraparticle diffusion governs adsorption rates. As theory [71] assumes, the PFO model describes physisorption, specifically the weak interaction between adsorbents and NSAIDs, while the PSO model refers to chemisorption, which explains the formation of a chemical bond between the adsorbent and pollutants [72]. Table 3 shows the kinetic parameters for PFO, PSO and IPD models during DCF and KTP adsorption. According to the high correlation coefficient (*R*^2^), the PSO kinetic model fitted better to the experimental data, so a new chemical bond was formed between the adsorbent materials and NSAIDs. Therefore, this agreement indicates that the PSO model accurately describes the adsorption kinetics, implying that a new chemical bond developed between the adsorbents and pharmaceutical compounds. In addition, the IPD model showed relatively low R^2^ values (0.380–0.788), indicating that intraparticle diffusion is not the sole rate-limiting step. The non-zero intercept (C ≠ 0) further confirms the involvement of film diffusion [73]. Moreover, the variation in C values among the different composites suggests that the significance of external resistance varies depending on their morphology and porosity, providing a complementary perspective on the kinetic processes beyond the dominant chemisorption.

#### 3.2.3. Isotherm Study

**Figure 10:**
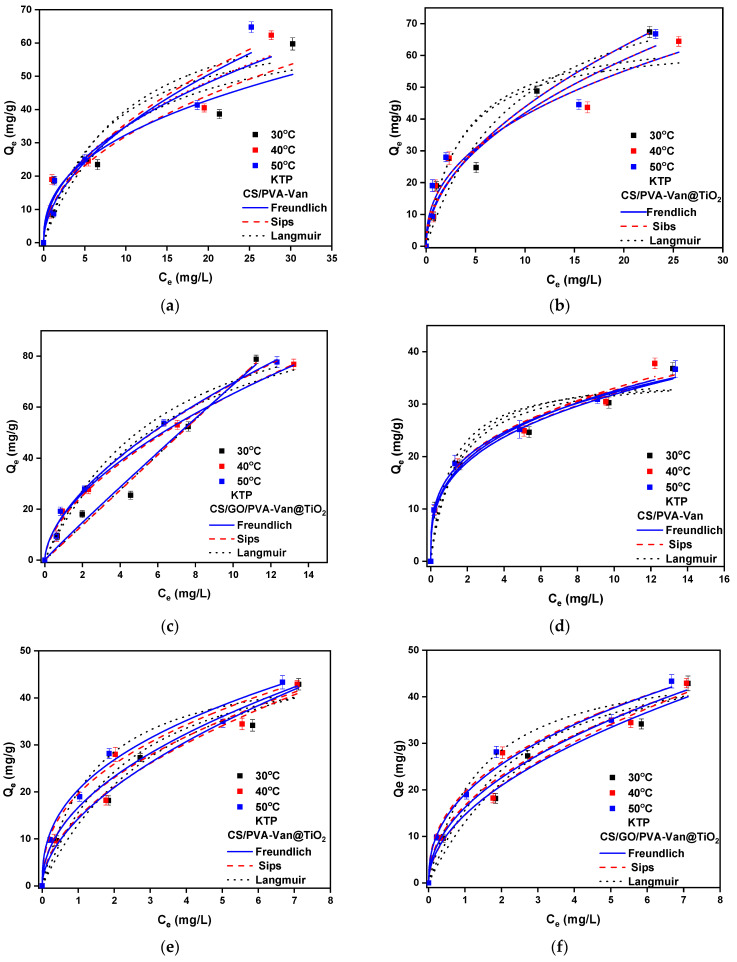
Isotherm models for the evaluation of removal efficiency of (**a**–**c**) DCF and (**d**–**f**) KTP using the new composites. Experimental conditions: for DCF removal C_0_ = 10–90 mg/L, pH = 5.0, T = 303, 313 and 323 K, t = 60 min; for KTP removal C_0_ = 10–20 mg/L, pH = 5.0, T = 303, 313 and 323 K, t = 60 min.

The adsorption equilibrium data were evaluated using three commonly applied isotherm models: the Freundlich, Langmuir and Sips models. The adsorption isotherms of DCF and KTP onto the new composites are shown in Figure 10, and the associated isotherm parameters are summarized in Table 4. In the case of CS/GO/PVA-VAN@TiO_2_, the adsorption of DCF and KTP is better fitted to the Freundlich isotherm model, as indicated by the higher correlation coefficient (*R*^2^ = 0.995 and 0.982 for DCF and KTP, respectively), in comparison to the Langmuir model (*R*^2^ = 0.986 and 0.970 for DCF and KTP, respectively). This fact means that the adsorption of the pharmaceutical compounds is multilayer on a heterogeneous surface area [74]. The Sips model also provides a satisfactory fit to the experimental data, reflecting its capability to account for heterogenous adsorption behavior and bridging characteristics between the Langmuir and Freundlich isotherms. According to the Freundlich isotherm model, the value of *n* provides insight into the nature of the adsorption process: when *n* < 1 the adsorption is chemical; when *n* = 1 it is linear; and when *n* > 1 the adsorption is a physical process [75]. Table 2 presents the 1/*n* values, which are less than one, confirming that the efficient NSAID removal from the new composite materials is a physical process.

#### 3.2.4. Thermodynamic Studies

In Table 5 are presented the thermodynamic parameters during pharmaceutical compound adsorption. The Gibbs free energy (Δ*G*^0^) was calculated at 303, 313 and 323 K at pH 5.0. The results affirmed that the adsorption of both DCF and KTP was spontaneous onto all of the new composite materials, which indicates the strong interactions between the pharmaceutical molecules and the adsorbent surface. Moreover, the positive value of Δ*H^0^* confirms that this process is an endothermic phenomenon [76]. This endothermic behavior can be attributed to the uptake of energy upon the formation of stable interactions between the NSAIDs and the active sites of the composites, indicating that the adsorption process was not driven by strong binding forces at the solid–solution interface [77].

Furthermore, a positive Δ*S*^0^ value demonstrated an increase in the disorder of the adsorbed molecules on the surface [78]. In the case of DCF and KTP adsorption, however, Δ*S*^0^ decreases at 313 K, indicating a reduction in molecular disorder. This behavior is consistent with a chemisorption mechanism [79]. The positive value of Δ*S*^0^ suggested an increase in randomness at the solid–solution interface during adsorption. This was probably caused by the displacement of water molecules or ions previously associated with the adsorbent surface or the hydration shell of the pharmaceutical molecules. As a result, the overall spontaneity of the process is enhanced.

#### 3.2.5. Effect of Mass

**Figure 11:**
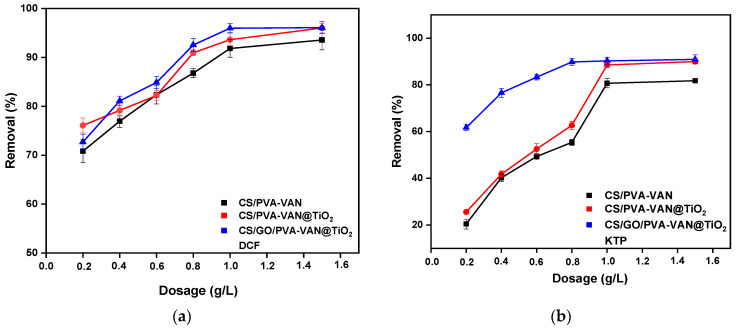
Effect of mass for (**a**) DCF and (**b**) KTP adsorption. (*C*_0_ = 50 mg/L, pH 5.0, t = 60 min, T = 303 K).

To determine the minimum amount of adsorbent mass by which the highest adsorption capacity was achieved, the effect of mass was studied in the range of 0.2–1.5 g/L. Figure 11 presents the experimental results. Initially, increasing the mass enhances the number of available active sites, improving the adsorption process. All of the new composites at a concentration of 1 g/L showed a high removal efficiency for NSAIDs, with the CS/GO/PVA-VAN@TiO_2_ composite exhibiting the highest performance. Overall, understanding this parameter is crucial not only for elucidating the adsorption mechanism and kinetics but also for the economical and practical design of adsorption systems, ensuring efficient material use while maintaining high removal efficiency.

### 3.3. Regeneration

**Figure 12:**
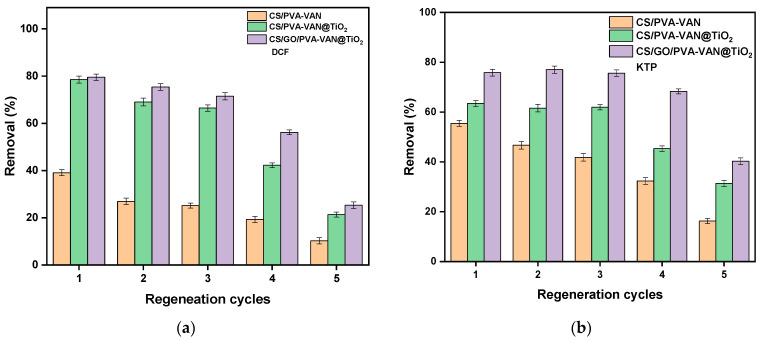
Adsorption of (**a**) DCF and (**b**) KTP for 5 cycles after regeneration at pH level 10 using 0.1 M NaOH treatment. (C_0_ = 50 mg/L, pH 5.0, t = 60 min, T = 303 K).

The regeneration ability of adsorbents constitutes a critical parameter in assessing their operational performance and long-term applicability in adsorption processes [80]. Figure 12 presents the regeneration results of the three adsorbent materials, i.e., CS/PVA-VAN, CS/PVA-VAN@TiO_2_ and CS/GO/PVA-VAN@TiO_2_ for DCF and KTP removal, after five regeneration cycles.

The reusability of all adsorbents for the removal of DCF and KTP was evaluated through successive adsorption–desorption experiments. During the regeneration process, a 0.01 M NaOH solution was employed to desorb the pharmaceutical compounds from the adsorbent surfaces. According to the literature, regeneration experiments have demonstrated that alkaline conditions are effective in promoting the desorption of pharmaceutical compounds.

**Figure 13:**
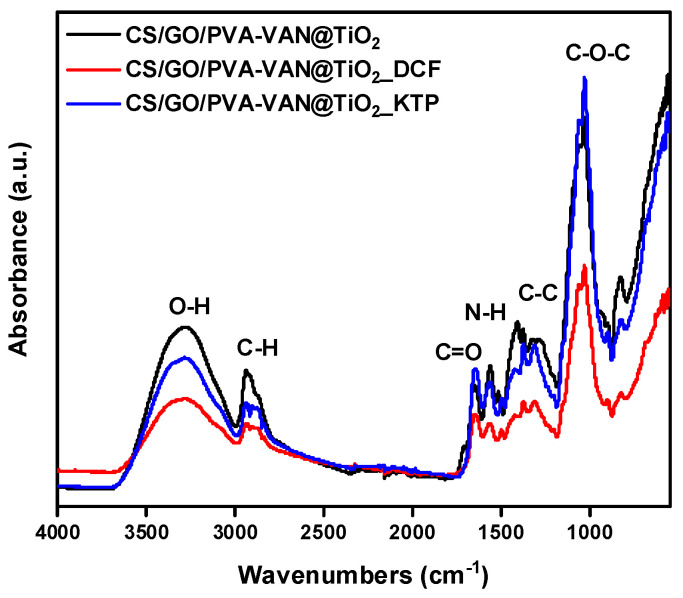
FTIR spectrum of optimum composite after five regeneration cycles during DCF and KTP removal.

In the 1st cycle, CS/GO/PVA-VAN@TiO_2_ removed 80% of DCF and 78% of KTP respectively. However, after five regeneration cycles the removal percentage for the material decreased to 25% and 40% for DCF and KTP, respectively. Therefore, reduction in the removal efficiency of DCF and KTP after five regeneration cycles is probably due to gradual changes in the structure and surface chemistry of the adsorbent material [81]. However, repeated washing with the alkaline solution during regeneration may cause partial damage or alteration of the active sites responsible for adsorption [82]. In addition, some pharmaceutical molecules may remain strongly attached to the surface through irreversible interactions, blocking access to adsorption sites. As a result, the material becomes less effective with each regeneration cycle. The deactivation of the optimal adsorbent after five regeneration cycles is evidenced by the FTIR spectrum (Figure 13).

The FTIR spectrum obtained after regeneration exhibited a reduction in the intensity of the characteristic peaks, accompanied by minor shifts. These observations suggest pore blockage, leading to a decrease in the adsorption capacity of the material after repeated regeneration cycles [83]. Furthermore, structural alterations were detected, indicating a potential loss of material stability [84].

### 3.4. Effect of Swelling

**Figure 14:**
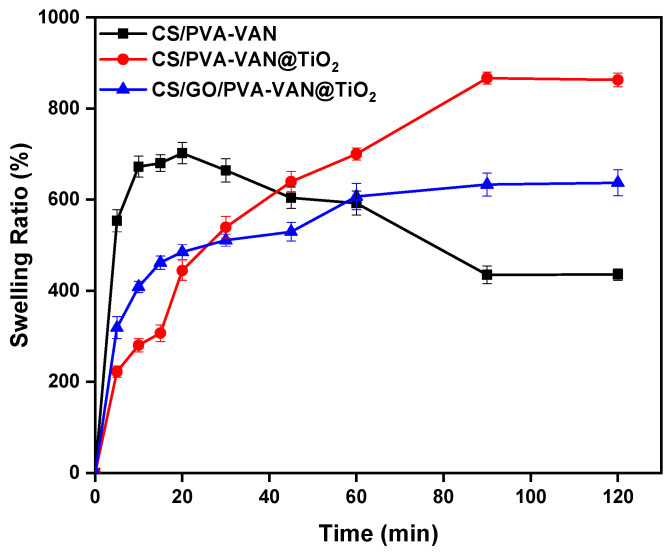
Swelling ratio of CS/PVA-VAN, CS/PVA-VAN@TiO_2_ and CS/GO/PVA-VAN@TiO_2_ (aquatic solution, pH 5.0, T = 303 K, t = 5–120 min).

Swelling, in accordance with kinetics, is an essential parameter which combines mass transport and mechanical deformation [85]. Chitosan, vanillin and GO contain various functional groups, such as animo groups and hydroxylic, carboxylic, and epoxy groups, which can be protonated in different pH values. A swelling study allows to understand the amount of water that could be retained inside the chemical structure of the adsorbents. Figure 14 shows the swelling ratio of adsorbents at pH 5.0 during a time interval of 5–120 min.

Initially, the CS/PVA-VAN composite exhibited a rapid increase in its swelling ratio during the first 20 min, indicating intense water absorption. Nevertheless, after this initial phase, the material gradually lost its ability to retain water, probably due to a limited availability of hydrophilic sites and partial disruption of the polymeric network, which led to partial structural degradation. On the other hand, the CS/GO/PVA-VAN@TiO_2_ composite exhibited a significantly higher swelling ratio compared to CS/PVA-VAN@TiO_2_ within the time interval of 5 to 60 min. This enhanced water uptake can be attributed to the presence of GO, which contains abundant functional groups capable of forming hydrogen bonds with water molecules [86], thereby promoting stronger intermolecular interactions and increasing the swelling capacity. This finding is significant because CS/GO/PVA-Van@TiO_2_ can swell and retain water within its structure without disturbing its chemical integrity. In this way, the contact between the pollutants and the pores of the adsorbent increased, thereby enhancing the adsorption process [87]. Following the 60 min measurement, the swelling ratio of CS/GO/PVA-VAN@TiO_2_ stabilized. This suggests that the incorporation of GO contributed to regulating water uptake and retention, resulting in a more stable and compact chemical structure [88].

### 3.5. Stability Test

**Figure 15:**
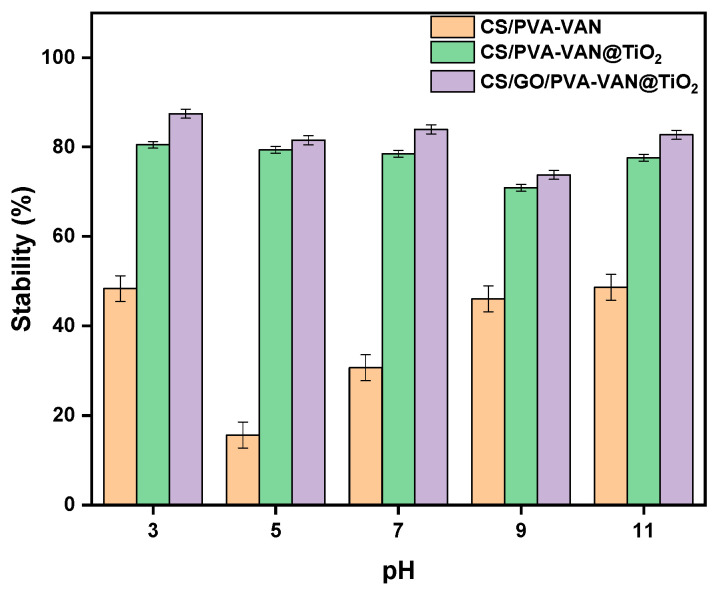
Stability study of CS/PVA-VAN, CS/PVA-VAN@TiO_2_ and CS/GO/PVA-VAN@TiO_2_ (aquatic solution, pH 3.0–11.0, t = 24 h, T = 303 K).

In Figure 15, the results of the stability studies of the new composites at different pH values are presented. Experimental data showed that the CS/GO/PVA-VAN@TiO_2_ composite presented the highest stability, especially at pH 5.0. The improved stability can be attributed to the incorporation of GO, which contains various useful functional groups that form strong hydrogen bonds and π-π interactions with the polymeric matrix, reinforcing the network and increasing mechanical integrity. Moreover, GO was incorporated into the chemical structure of the polymeric matrix (view 3.1.3) and formed strongly chemical bonds which did not allow the decomposition of material [89]. On the other hand, CS/PVA-VAN and CS/PVA-VAN@TiO_2_ degraded 30% and 80%, respectively, under experimental conditions in an aquatic solution (pH 3.0–11.0, t = 24 h, T = 303 K), probably due to weaker polymeric interactions and the absence of stabilizing GO. Consequently, the presence of GO strengthens the network through strong hydrogen bonding and electrostatic interactions with the CS and PVA chains, resulting in a dense and highly interconnected polymeric structure that restricts water penetration. In the absence of GO, the network exhibits a lower degree of crosslinking and demonstrates increasing susceptibility to hydrolytic cleavage of the Schiff base bonds. Overall, these findings indicate that GO incorporation not only improves structural integrity but enhances the composite’s suitability for practical applications in wastewater treatment.

### 3.6. Binary System

**Figure 16:**
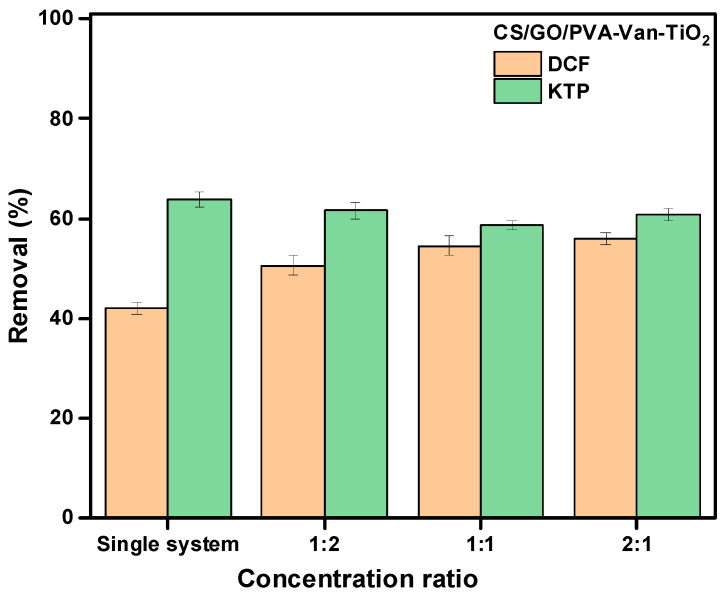
Adsorption study on the removal efficiency of DCF and KTP from a binary system using the CS/GO/PVA-Van@TiO_2_ composite.

The adsorption process of DCF and KTP was investigated in a binary system at different molar ratios (1:2, 1:1 and 2:1) tο evaluate the interactions between the two compounds. According to the literature, a synergistic effect occurs when the presence of one compound enhances the adsorption of another, whereas a competitive effect arises when the compounds compete for the same adsorption sites on the adsorption surface. As shown in Figure 16, the presence of KTP enhances the adsorption of DCF in all ratios, as reflected by the increased removal efficiency of DCF (R%) from 42% to ≈55%, indicating a synergistic effect. Conversely, KTP adsorption was slightly suppressed in the presence of DCF, with removal efficiency reduced from 63% to 60%.

However, the selectivity coefficient values of 0.80, 0.84 and 0.82 at different concentration ratios (1:2, 1:1 and 2:1, respectively) suggest that the adsorbent exhibited a higher preference for KTP (Table 6). Although both molecules contain aromatic rings and a carboxylic group and therefore may compete for common active sites (e.g., amino and hydroxyl groups of CS and oxygenated groups of GO), differences in their molecular structure and hydrophobicity influence their adsorption behavior. Additionally, KTP, which possesses a benzophenone moiety and a more accessible carbonyl/carboxyl group, can interact more effectively with the polar functional groups of the CS/GO matrix though hydrogen bonding and electrostatic interactions, leading to a slightly higher affinity of the adsorbent for KTP [90]. On the other hand, DCF, which is bulkier and more hydrophobic due to its two chlorinated aromatic rings, is more likely to interact with the graphitic domains of GO via hydrophobic and π-π* interactions [91]. These differences help explain the observed selectivity toward KTP (S < 1), while the presence of KTP may simultaneously facilitate additional adsorption of DCF in binary system (Ri > 1), suggesting a combination of competitive and cooperative adsorption mechanisms. Specially, the synergistic effect can be attributed to a pre-adsorption mechanism, in which KTP (S < 1) initially occupies adsorption sites and modifies the local surface environment, creating more favorable conditions for subsequent DCF uptake (e.g., via π-π* stacking interactions, hydrogen bonding or changes is surface polarity). Similar behavior has been recently reported by Li et al., where pre-adsorption of aromatic compounds in MIL-53 (AI) systems promotes complementary or synergistic co-adsorption via π-π stacking, hydrophobic interactions, and the formation of additional adsorption sites [92]. This supports that the observed behavior cannot explained solely by competitive adsorption, but rather involves a surface “activation” effect induced by the initially adsorbent molecule.

## 4. Conclusions

In this study, three new composite materials, CS/PVA-VAN, CS/PVA-VAN@TiO_2_ and CS/GO/PVA-VAN@TiO_2_, were synthesized to remove DCF and KTP from aquatic solutions. Crosslinking was achieved with GLA and PVA. All new adsorbents are characterized via FTIR, XRD, SEM and BET techniques. According to BET analysis, the surface area of CS/GO/PVA-Van@TiO_2_ (64.86 m^2^/g) is approximately double that of CS/PVA-Van. Furthermore, the XRD techniques verify that GO decreases the crystallinity of the composite and it is dispersed inside the polymeric network. Moreover, according to batch experiments, the optimum pH value is 5.0 for both DCF and KTP, while the kinetic data fit better to the PSO kinetic model. Therefore, the isotherm study demonstrates that Freundlich model better describes the adsorption of DCF and KTP by all of the new composites, and the adsorption capacity of CS/GO/PVA-VAN@TiO_2_ is 114.53 mg/g and 65.20 mg/g for DCF and KTP, respectively. According to the thermodynamic parameters, NSAID adsorption is endothermic and spontaneous for all adsorbents. In addition, the regeneration study demonstrates that the optimum CS/GO/PVA-VAN@TiO_2_ composite material retains its removal efficiency for up to five consecutive cycles, indicating its satisfactory reusability for the adsorption of DCF and KTP from aqueous solutions. Moreover, the addition of GO to the polymeric matrix enhances its hydrophilicity, stability and mechanical properties, as confirmed by the swelling and stability studies. Finally, in the binary system, KTP is preferentially adsorbed, while its presence simultaneously enhances DCF adsorption, indicating a synergistic effect despite selectivity of KTP.

## Figures and Tables

**Table 1:** Synthesis of CS/PVA composites with VAN, TiO_2_ and GO (final volume 100 mL).

Adsorbent	PVA (g)	Van (g)	CS (g)	TiO_2_ (g)	GO (g)	Acetic Acid (mL)	GLA (mL)
CS/PVA-VAN	0.5	0.2	1.0	-	-	2	2
CS/PVA-VAN@TiO_2_	0.5	0.2	1.0	0.2	-	2	2
CS/GO/PVA-VAN@TiO_2_	0.5	0.2	1.0	0.2	0.01	2	2

**Table 2:** BET parameter of new composite adsorbent materials.

Adsorbent Material	Surface Area (m^2^/g)	Pore Volume (cm^3^/g)	Average Pore Diameter (nm)
CS/PVA-Van@TiO_2_	32.46	0.029	161.180
CS/GO/PVA-Van@TiO_2_	64.86	0.060	2.016

**Table 3:** Kinetic data of PFO, PSO and IPD kinetic models for DCF and KTP.

Pollutant	Adsorbent	*Q_e,exp_* (mg/g)	PFO			PSO			IPD		
			*K*_1_ (1/min)	*Q_e,cal_* (mg/g)	*R* ^2^	*K*_2_ (L/mg.min)	*Q_e,cal_* (mg/g)	*R* ^2^	*K_IPD_* (mg* min^−0.5^/g)	*C* (mg/g)	*R* ^2^
DCF	CS/PVA-VAN	47.21	0.092	45.41	0.985	0.003	42.24	0.987	3.33	12.32	0.788
	CS/PVA-VAN@TiO_2_	49.16	0.205	45.68	0.978	0.007	48.35	0.995	2.88	21.68	0.588
	CS/GO/PVA-VAN@TiO_2_	49.20	0.335	46.47	0.991	0.016	47.99	0.998	3.20	18.58	0.568
KTP	CS/PVA-VAN	46.16	0.103	42.85	0.984	0.003	46.20	0.979	2.75	16.94	0.670
	CS/PVA-VAN@TiO_2_	46.64	0.431	44.62	0.977	0.024	46.42	0.993	2.26	28.42	0.380
	CS/GO/PVA-VAN@TiO_2_	47.68	0.366	46.24	0.993	0.020	47.69	0.995	2.78	25.53	0.444

**Table 4:** Isotherm parameters of the Langmuir, Freundlich and Sips models for the DCF and KTP adsorptions.

Langmuir model				
Pollutant	Adsorbent	K_L_ (L/mg)	*Q_m_* (mg/g)	R^2^
DCF	CS/PVA-VAN	0.102	68.60	0.876
	CS/PVA-VAN@TiO_2_	0.108	91.19	0.945
	CS/GO/PVA-VAN@TiO_2_	0.141	114.53	0.986
KTP	CS/PVA-VAN	0.706	36.07	0.935
	CS/PVA-VAN@TiO_2_	0.289	58.51	0.974
	CS/GO/PVA-VAN@TiO_2_	0.275	65.20	0.970
Freundlich model				
Pollutant	Adsorbent	K_LF_ (L/g)	1/n	R^2^
DCF	CS/PVA-VAN	10.867	0.47	0.939
	CS/PVA-VAN@TiO_2_	13.115	0.53	0.975
	CS/GO/PVA-VAN@TiO_2_	16.784	0.59	0.995
KTP	CS/PVA-VAN	15.165	0.32	0.990
	CS/PVA-VAN@TiO_2_	14.732	0.52	0.982
	CS/GO/PVA-VAN@TiO_2_	15.362	0.56	0.982
Sips model				
Pollutant	Adsorbent	K_F_ (mg/g)(L/mg)^1/n^	1/n	R^2^
DCF	CS/PVA-VAN	10.83	0.40	0.933
	CS/PVA-VAN@TiO_2_	10.62	0.52	0.975
	CS/GO/PVA-VAN@TiO_2_	9.80	1.02	0.981
KTP	CS/PVA-VAN	9.21	0.32	0.990
	CS/PVA-VAN@TiO_2_	8.31	0.55	0.987
	CS/GO/PVA-VAN@TiO_2_	8.20	0.51	0.982

**Table 5:** Thermodynamic parameters for DCF and KTP adsorption.

Material	*T* (K)	Δ*G*^0^ (kJ/mol)	Δ*H*^0^ (kJ/mol)	Δ*S*^0^ (kJ/mol∙K)	*R* ^2^
	DCF				
CS/PVA-VAN	303	−0.72	8.62	0.032	0.977
	313	−1.13			
	323	−1.35			
CS/PVA-VAN@TiO_2_	303	−1.50	7.26	0.030	0.941
	313	−1.83			
	323	−2.09			
CS/GO/PVA-VAN@TiO_2_	303	−4.56	8.27	0.042	0.923
	313	−5.03			
	323	−4.34			
	KTP				
CS/PVA-VAN	303	−2.50	9.01	0.039	0.996
	313	−2.84			
	323	−3.28			
CS/PVA-VAN@TiO_2_	303	−3.35	5.40	0.030	0.976
	313	−3.71			
	323	−3.94			
CS/GO/PVA-VAN@TiO_2_	303	−4.37	2.86	0.025	0.990
	313	−4.54			
	323	−4.87			

**Table 6:** Adsorption characteristics of DCF and KTP in binary systems at different concentration ratios.

System DCF:KTP	NSAIDs	C_0_ (mg/L)	C_e_ (mg/L)	Q_e_ (mg/g)	S_DCF/KTP_	R_i_
1:2	DCF	25	10.94	14.06	0.80	1.77
	KTP	50	19.20	29.38		0.91
1:1	DCF	50	22.71	27.29	0.84	1.65
	KTP	50	20.62	29.38		0.81
2:1	DCF	50	22.00	28.00	0.82	1.75
	KTP	25	9.82	15.18		0.88

## Data Availability

The data will be made available on request.
